# Middle Eastern male ACL‐deficient patients exhibit steeper posterior tibial slope than European counterparts: A cross‐cultural matched cohort study

**DOI:** 10.1002/jeo2.70790

**Published:** 2026-06-10

**Authors:** Iacopo Romandini, Khalid AlKhelaifi, Luca Macchiarola, Bashir Ahmed Zikria, Caroline Mouton, Romain Seil, Alan Getgood

**Affiliations:** ^1^ Orthopaedic Surgery Department Aspetar Orthopedic and Sports Medicine Hospital Doha Qatar; ^2^ Department of Orthopedics and Traumatology Fondazione IRCCS Casa Sollievo della Sofferenza San Giovanni Rotondo Italy; ^3^ Department of Orthopaedic Surgery The Johns Hopkins University School of Medicine Baltimore Maryland USA; ^4^ Department of Orthopaedic Surgery Centre Hospitalier Luxembourg—Clinique d'Eich Luxembourg Luxembourg; ^5^ Luxembourg Institute of Research in Orthopaedics Sports Medicine and Science (LIROMS) Luxembourg Luxembourg; ^6^ Human Motion, Orthopaedics, Sports Medicine and Digital Methods (HOSD) Luxembourg Institute of Health (LIH) Luxembourg Luxembourg

**Keywords:** anterior cruciate ligament (ACL), knee, Middle East, posterior tibial slope (PTS)

## Abstract

**Purpose:**

The posterior tibial slope (PTS) is a key anatomical factor influencing anterior cruciate ligament (ACL) stability. Steeper slopes have been associated with increased anterior tibial translation (ATT) and a higher risk of graft failure following ACL reconstruction. Ethnic and regional differences in tibial morphology have been suggested, but comparative data across populations remain limited. This study aimed to compare medial PTS angles in ACL‐deficient male patients from Europe and the Middle East.

**Methods:**

Ninety‐four male patients undergoing primary ACL reconstruction (47 from Europe, 47 from the Middle East) were retrospectively included and matched 1:1 for age and sex. PTS was measured on true lateral radiographs using a validated method. Intra‐ and inter‐rater reliability were assessed with intraclass correlation coefficients (ICCs).

**Results:**

The two cohorts were homogeneous in terms of key demographic variables. Surgical preferences differed significantly between groups in graft type (*p* < 0.0001) and meniscal repair rates (*p* = 0.002), with European patients more frequently undergoing meniscal procedures. However, the use of lateral extra‐articular tenodesis was similar between groups (*p* = 0.80). PTS measurements showed good to excellent reliability (ICC 0.79–0.98). The Middle Eastern cohort had a significantly higher mean PTS than the European group (12.9 ± 3.4° vs. 10.3 ± 3.8°, *p* = 0.002). A larger proportion of Middle Eastern patients exceeded the 12° threshold considered biomechanically relevant (46.8% vs. 25.5%, *p* = 0.04; odds ratio = 2.59). No significant difference was observed in bone marrow oedema (*p* = 0.42).

**Conclusions:**

ACL‐deficient male patients from the Middle East exhibited significantly steeper PTS angles compared to their European counterparts, with a higher proportion exceeding 12°. This anatomical difference reflects regional morphotype characteristics within the studied cohorts and highlights potential variability in tibial slope distribution across populations.

**Level of Evidence:**

Level III, retrospective matched cohort study.

**Abbreviations**
ACL: anterior cruciate ligament
ACLR: anterior cruciate ligament reconstruction
ACWHTO: anterior closing‐wedge high tibial osteotomy
ATT: anterior tibial translation
BTB: bone–patellar tendon–bone
CI: confidence interval
ICC: intraclass correlation coefficient
IQR: interquartile range
LET: lateral extra‐articular tenodesis
LM: lateral meniscus
MM: medial meniscus
MRI: magnetic resonance imaging
NE: not estimable
OR: odds ratio
PTS: posterior tibial slope
SD: standard deviation
SEM: standard error of measurement
STG: semitendinosus–gracilis graft

## INTRODUCTION

The posterior tibial slope (PTS), defined as the angle between the tibial longitudinal axis and the posterior inclination of the tibial plateau [13], plays a critical role in maintaining sagittal plane stability of the knee [20]. In anterior cruciate ligament (ACL)–deficient knees, an increased PTS has been associated with greater anterior tibial translation (ATT), altered joint mechanics and a higher risk of instability or graft failure following reconstruction [11, 18, 19, 30]. Several biomechanical studies have demonstrated that a steeper medial or lateral slope amplifies anterior shear forces on the tibia, particularly under weight‐bearing conditions, thus increasing stress on the ACL or its graft [10, 21, 25, 31]. As a result, PTS has gained attention primarily as a biomechanical marker associated with ACL injury and graft failure risk [35, 44]. Although tibial slope can be surgically modified in selected cases (e.g., slope‐reducing osteotomy), it is generally considered a non‐modifiable anatomical parameter in routine clinical pathways. However, published thresholds for ‘at‐risk’ slopes vary, and measurement methods differ across studies, reflecting not only methodological inconsistencies but also population‐specific morphological traits of the tibial plateau that may limit comparability and consensus regarding clinical indications [15, 40, 42].

Population‐based differences in PTS appear to follow patterns influenced by ethnic background, skeletal morphology and, possibly, lifestyle‐related factors [8]. Radiological studies from various regions worldwide have reported population‐specific PTS values, often differing significantly from those observed in European cohorts [2, 7, 26, 27, 28]. In particular, Middle Eastern populations have been suggested to exhibit higher average PTS angles, although existing results remain heterogeneous and are mostly derived from single‐centre studies using non‐uniform methods [1, 2]. These variations may reflect both genetic determinants and functional adaptations to daily postural habits, such as kneeling or squatting, which are more prevalent in certain cultures [26]. Despite these insights, no study to date has directly compared PTS distributions between Middle Eastern and European patients within a homogeneous cohort of ACL‐deficient individuals. Understanding such anatomical differences may contribute to a better characterization of population‐specific knee morphology in ACL‐deficient individuals.

The present study aimed to investigate and compare PTS values in ACL‐deficient male patients from two distinct regions: Europe and the Middle East. The hypothesis was that the Middle Eastern cohort would demonstrate significantly steeper PTS angles than their European counterparts.

## METHODS

### Study design and ethics

This study was designed as a retrospective cohort study conducted on data extracted from medical records obtained for clinical purposes at two specialized sports medicine centres: the Centre Hospitalier de Luxembourg, Clinique d'Eich, Luxembourg, and the Aspetar Orthopaedic and Sports Medicine Hospital, Doha, Qatar. Ethical approval was obtained from the Institutional Review Board of the Aspetar Orthopaedic and Sports Medicine Hospital, Doha, Qatar (Protocol registration: X202209041). Only patients who had provided written consent for the scientific use of their clinical data were included. Data were retrospectively extracted from medical records obtained for standard clinical purposes, anonymized prior to analysis and handled by the clinical teams directly responsible for patient care, who had authorized access to the records. Patient confidentiality was strictly maintained, and no additional procedures, interventions or risks beyond standard clinical practice were introduced.

### Study population

#### European group

All consecutive male patients presenting at the Centre Hospitalier de Luxembourg for primary ACL deficiency confirmed by both magnetic resonance imaging (MRI) and clinical examination were considered eligible, starting from January 2019 until December 2021.

Inclusion criteria were:

1. Nationality from a European Union country.
2. Availability of a true lateral radiograph of the affected knee showing perfect superimposition of the posterior femoral condyles and at least 15 cm of visible tibial shaft.

Exclusion criteria included:

1. Insufficient image quality (condylar superimposition >3 mm or <15 cm of tibial shaft visible).
2. Previous surgical procedures on the ACL‐deficient knee.
3. Open growth plates (age <16 years).

#### Middle Eastern group

To obtain a 1:1 age (±2 years) and sex‐matched comparison, all consecutive male patients evaluated at the Aspetar Orthopaedic and Sports Medicine Hospital for primary ACL deficiency confirmed by MRI and clinical examination were considered eligible. Inclusion and exclusion criteria were identical to the European cohort, except that eligible patients were required to have nationality from one of the Middle Eastern countries.

### Data collection

For all included patients, the following variables were extracted: date of birth, age at radiograph and intraoperative reports when available. MRI scans were reviewed to confirm the presence of ACL rupture and to document concomitant findings. Radiological assessments were first verified according to official radiology reports and then independently reviewed by a dedicated researcher (L. M. for the European group and I. R. for the Middle Eastern group). In case of discrepancies between the radiology report and the independent review, a third blinded assessment was performed by a senior orthopaedic surgeon (R. S. for the European group and K. A. K. for the Middle Eastern group).

### Radiographic assessment

Radiographic measurements were performed on true short lateral radiographs of the affected knee, obtained with approximately 15°–20° of flexion and perfect posterior condyle superimposition. Radiographic acquisition protocols followed standardized positioning criteria across both centres to ensure comparability of measurements. Radiographs not meeting predefined quality criteria (including inadequate condylar superimposition or insufficient visualization of the tibial shaft) were excluded from analysis.

**Figure 1:**
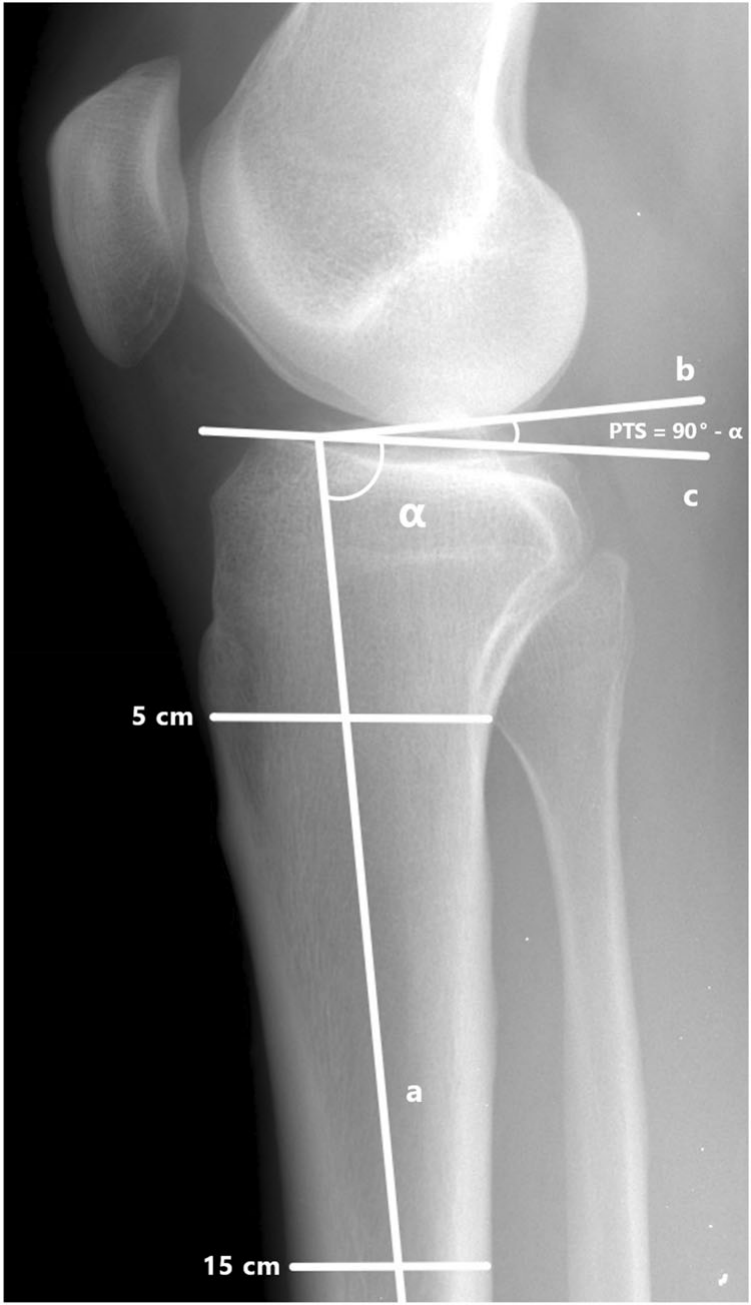
Lateral radiograph of an affected knee at 15°–20° of flexion. Posterior tibial slope (PTS = 90° − *α*) is measured between the perpendicular to the tibial axis (b), defined as the line passing through two points (a), both equidistant from the anterior and posterior tibial cortex (5 and 15 cm distal to the tibial plateau), and the tangent to the anterior and posterior edges of the medial tibial plateau (c).

The PTS was measured on the medial tibial plateau according to the method described by Dejour and Bonnin [13], corresponding to the ‘angle *α*’. On a true lateral radiographic view, the PTS was defined as the acute angle between the line tangent to the anterior and posterior margins of the medial tibial plateau and a line perpendicular to the proximal tibial anatomical axis. The tibial axis was determined by connecting the midpoints between the anterior and posterior cortices of the tibial diaphysis, identified at 5 and 15 cm distal to the joint line. The final PTS value was expressed as (90° − *α*), yielding a positive slope for posterior inclination (Figure 1).

To assess measurement reliability, all radiographs were analysed twice by the same investigator (I. R.), 1 week apart, to calculate intra‐rater reliability. Two additional independent observers, L. M. for the European group and K. A. K. for the Middle Eastern group, performed the same measurements to determine inter‐rater reliability. All assessors were blinded to cohort allocation during the measurement process.

### Statistical analysis

A priori power analysis (comparison of two independent means) indicated that a minimum of 42 patients per group was required to detect a clinically meaningful difference of 2° in PTS between groups, with a standard deviation (SD) of 3.2°, *α* = 0.05, and power = 0.80. Continuous variables (e.g., age, PTS) were assessed for normality using the Kolmogorov–Smirnov test and presented as mean ± SD or as median with interquartile range (IQR) when non‐normally distributed. Categorical data (e.g., meniscal lesions) were expressed as numbers and percentages. Patients were matched 1:1 between cohorts for age (±2 years) and sex during the selection process to obtain comparable groups. Continuous variables were compared using the independent *t* test when normally distributed and the Mann–Whitney *U* test when non‐normally distributed. Categorical variables were compared using the *χ* ^2^ test or Fisher's exact test when appropriate. Odds ratios (ORs) with 95% confidence intervals (95% CIs) were calculated using univariable logistic regression models to estimate the association between group allocation (Middle East vs. Europe) and categorical outcomes. Intra‐ and inter‐rater reliability for PTS measurements were assessed using the intraclass correlation coefficient (ICC) with a two‐way mixed‐effects model for absolute agreement. Reliability was classified as: poor (ICC < 0.50), moderate (0.50–0.75), good (0.75–0.90) or excellent (>0.90). The standard error of measurement (SEM) was also reported. The significance threshold was set at *p* < 0.05. All analyses were performed using MedCalc Software (Version 20.0; MedCalc Software).

## RESULTS

### Patients' characteristics

A total of 47 European patients met the inclusion criteria and were included in the study. They were successfully matched 1:1 with 47 Middle Eastern patients, resulting in two cohorts of equal size. Matching was excellent for the selected criteria (Table 1): all included patients were male, of comparable age distribution (26.6 ± 7.3 vs. 26.6 ± 7.3 years, *p* = 0.99), and presented with primary ACL tears confirmed by clinical examination and MRI. The side distribution was also similar between groups (59.6% right knees in the European group, 70.2% in the Middle Eastern group, *p* = 0.28), as well as athlete status (68.2% vs. 50.0%, *p* = 0.09).

**Table 1:** Baseline demographic characteristics of the matched cohort.

Characteristic	Europe (*n* = 47)	Middle East (*n* = 47)	OR (95% CI)	*p* value
Sex (male)	47 (100%)	47 (100%)	1.00 (NE)	1.00
Age (years)	26.6 ± 7.3	26.6 ± 7.3	1.00 (0.94–1.07)	0.99
Side (right)	28 (59.6%)	33 (70.2%)	1.60 (0.68–3.76)	0.28
Athletes	32 (68.2%)	23 (50.0%)	0.47 (0.19–1.13)	0.09

*Note*: Values are expressed as mean ± standard deviation for continuous variables and as number (%) for categorical variables. ORs are calculated for the Middle East group compared with the European group.

Abbreviations: CI, confidence interval; NE, not estimable; ORs, odds ratios.

**Figure 2:**
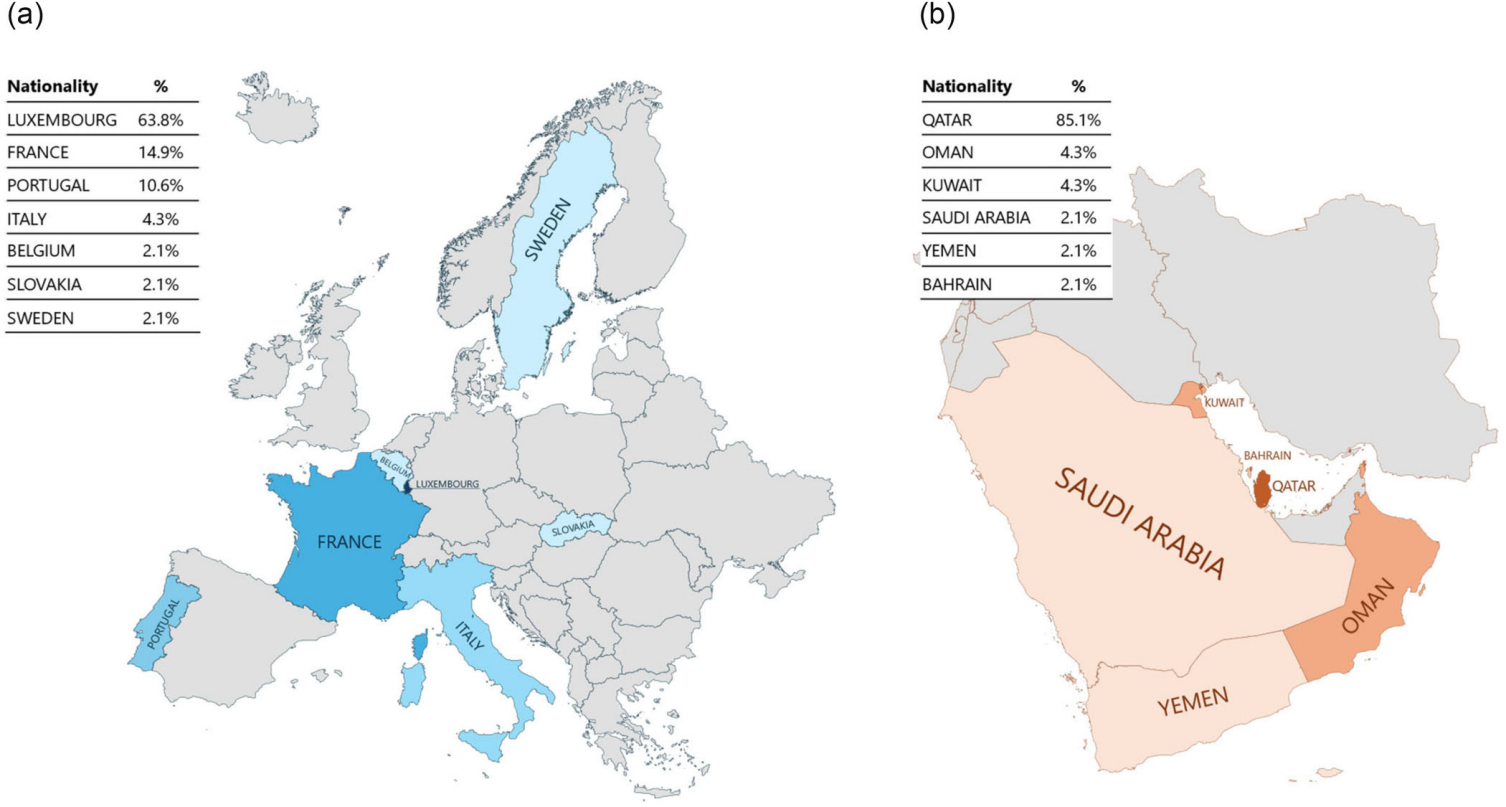
Geographic distribution of patients by nationality in the two study cohorts. (a) European group, showing the percentage distribution of patients across Luxembourg, France, Portugal, Italy, Belgium, Slovakia and Sweden. (b) Middle Eastern group, showing the percentage distribution across Qatar, Oman, Kuwait, Saudi Arabia, Yemen and Bahrain. Shading intensity represents the proportion of patients from each country within the respective cohort.

Regarding nationality, the European group was predominantly from Luxembourg (30/47, 63.8%), followed by France (*n* = 7), Portugal (*n* = 5), Italy (*n* = 2), and single cases from Belgium, Slovakia and Sweden. The Middle Eastern group was composed mainly of patients from Qatar (40/47, 85.1%), with additional patients from Oman (*n* = 2), Kuwait (*n* = 2), Saudi Arabia (*n* = 1), Yemen (*n* = 1) and Bahrain (*n* = 1) (Figure 2).

Regarding sports participation, the European cohort also showed football as the most represented discipline (46.8%), followed by basketball, running, handball and rugby. In contrast, the Middle Eastern cohort consisted predominantly of football players (44.7%), with additional participants engaged in basketball, volleyball, martial arts and various recreational activities, including paramotor sports.

### Surgical characteristics

Surgical details for both groups are reported in Table 2. A significant difference was observed in the graft selection between cohorts. In the European group, the quadriceps tendon was the predominant graft choice (59.6%), whereas hamstring and BTB grafts were used in 21.3% and 19.1% of cases, respectively (*p* < 0.0001). Conversely, in the Middle Eastern group, hamstring tendon (semitendinosus–gracilis graft [STG]) was the most frequently used graft (59.6%), followed by bone–patellar tendon–bone (BTB, 38.3%) and quadriceps tendon (2.1%). The use of a lateral extra‐articular tenodesis (LET) was similar between groups (21.3% vs. 19.1%, *p* = 0.80). However, a significantly higher rate of associated meniscal procedures was recorded in the European cohort (74.5% vs. 44.7%, *p* = 0.002). Specifically, medial meniscus repair (21.3% vs. 6.4%, *p* = 0.04) and lateral meniscus repair (36.2% vs. 12.8%, *p* = 0.01) were both more common among European patients. Differences in partial meniscectomies were not statistically significant for either the medial (14.9% vs. 6.4%, *p* = 0.19) or the lateral compartment (17.0% vs. 12.8%, *p* = 0.57).

**Table 2:** Surgical characteristics.

Characteristic	Europe (*n* = 47)	Middle East (*n* = 47)	OR (95% CI)	*p* Value
Graft type	STG 10 (21.3%) BTB 9 (19.1%) Quad 28 (59.6%)	STG 28 (59.6%) BTB 18 (38.3%) Quad 1 (2.1%)	NE	<0.0001
LET	10 (21.3%)	9 (19.1%)	0.88 (0.32–2.40)	0.80
Meniscal procedure	35 (74.5%)	21 (44.7%)	0.28 (0.12–0.65)	0.002
MM meniscectomy	7 (14.9%)	3 (6.4%)	0.39 (0.10–1.64)	0.19
LM meniscectomy	8 (17.0%)	6 (12.8%)	0.72 (0.23–2.27)	0.57
MM repair	10 (21.3%)	3 (6.4%)	0.25 (0.07–0.94)	0.04
LM repair	17 (36.2%)	6 (12.8%)	0.27 (0.09–0.77)	0.01

*Note*: Values are expressed as number (%) for categorical variables.

Abbreviations: ACLR, anterior cruciate ligament reconstruction; BTB, bone–patellar tendon–bone; CI, confidence interval; LET, lateral extra‐articular tenodesis; LM, lateral meniscus; MM, medial meniscus; NE, not estimable; OR, odds ratio; STG, semitendinosus–gracilis graft.

### Radiological results

The intra‐ and inter‐rater reliabilities of the radiographic measurements for PTS ranged from good to excellent in both cohorts. In the European group, intra‐rater reliability showed good agreement (ICC = 0.79, 95% CI 0.65–0.87) with a SEM of 1.2°, while inter‐rater reliability was also good (ICC = 0.83, 95% CI 0.76–0.88; SEM 1.0°). In the Middle Eastern group, both intra‐ and inter‐rater reliability demonstrated excellent agreement, with ICC values of 0.98 (95% CI 0.92–0.99; SEM 0.8°) and 0.81 (95% CI 0.48–0.92; SEM 1.9°), respectively.

A total of three radiographs per cohort were excluded due to insufficient image quality. The mean PTS was significantly higher in the Middle Eastern cohort compared with the European cohort (12.9 ± 3.4° vs. 10.3 ± 3.8°, *p* = 0.002). When applying a 12° cut‐off value, a greater proportion of patients in the Middle Eastern group exceeded this threshold (46.8% vs. 25.5%, *p* = 0.04; OR = 2.59, 95% CI 1.09–6.15), indicating a steeper PTS distribution in this population. Finally, the prevalence of bone marrow oedema did not differ significantly between groups (40.4% in the European group vs. 48.9% in the Middle Eastern group, *p *= 0.42; OR = 1.41, 95% CI 0.61–3.27). Radiological findings are summarized in Table 3.

**Table 3:** Radiological characteristics.

Characteristic	Europe (*n* = 47)	Middle East (*n* = 47)	OR (95% CI)	*p* Value
PTS (°)	10.3 ± 3.8	12.9 ± 3.4	1.27 (1.09–1.49)	0.002
PTS > 12°	12 (25.5%)	22 (46.8%)	2.59 (1.09–6.15)	0.04
Bone oedema	19 (40.4%)	23 (48.9%)	1.41 (0.61–3.27)	0.42

*Note*: Values are expressed as mean ± standard deviation for continuous variables and as number (%) for categorical variables.

Abbreviations: CI, confidence interval; OR, odds ratio; PTS, posterior tibial slope.

**Figure 3:**
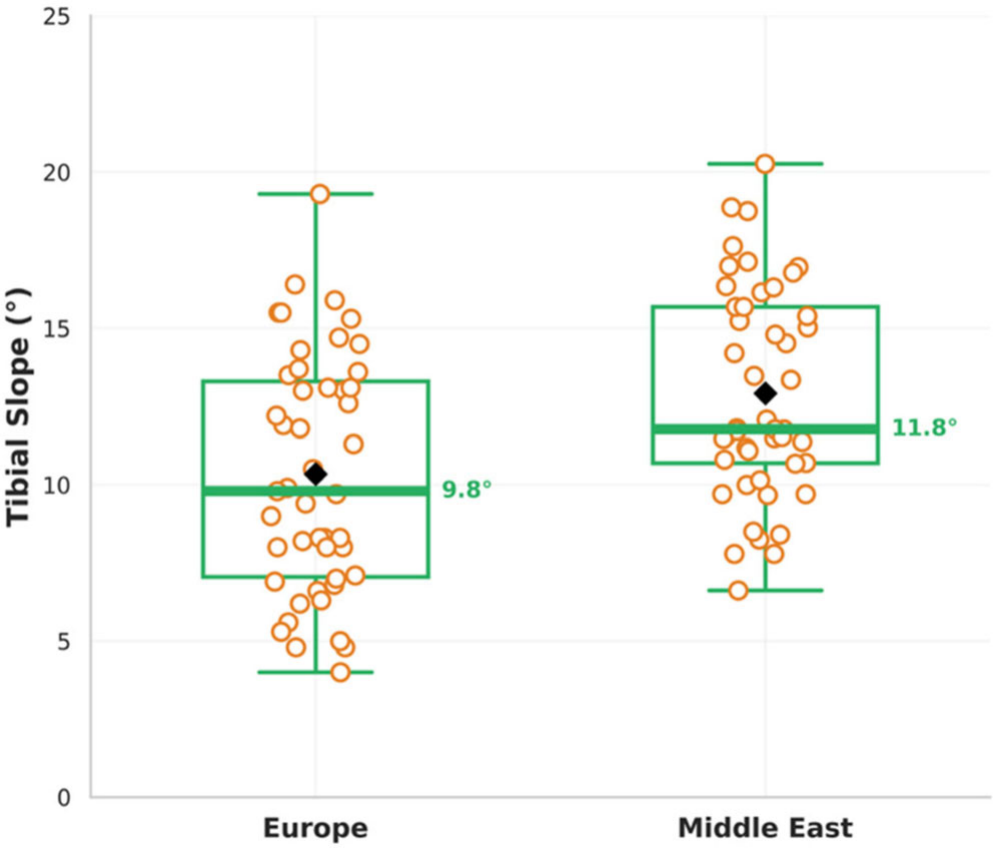
Distribution of PTS in European and Middle Eastern ACL‐deficient patients. Boxplots illustrate the distribution of PTS values in the European and Middle Eastern cohorts. The central green horizontal line represents the median value, while the box indicates the interquartile range and the whiskers the overall data spread. Individual patients are shown as hollow circles. Black diamond markers indicate the mean value for each group. The Middle Eastern cohort demonstrated a higher PTS compared with the European cohort. ACL, anterior cruciate ligament; PTS, posterior tibial slope.

This difference is visually represented in Figure 3, which illustrates the distinct distribution of PTS values between the two cohorts.

## DISCUSSION

The main finding of the present study is that ACL‐deficient male patients from the Middle East exhibited significantly steeper PTS angles compared to their European counterparts, with a higher proportion exceeding the 12° threshold.

This result stems from the rigorous methodological approach adopted in the study. Two well‐balanced cohorts of ACL‐deficient male patients from Europe and the Middle East were matched 1:1 based on key demographic variables. This strategy, combined with the multicenter design, improves comparability between cohorts and helps reduce potential confounding factors. Only male patients were included to limit sex‐related anatomical variability reported in previous literature [39]. To our knowledge, the literature lacks direct comparisons of PTS values between patients from different geographic or ethnic backgrounds. Most of the existing literature has consisted of single‐centre, observational studies that characterized PTS values within isolated populations, often relying on non‐standardized measurement techniques and lacking reference groups [2, 7, 27, 28]. Chiu et al. [7] analysed PTS in a Chinese cohort and reported values that differed from Western norms. Katchy et al. [27] conducted a similar investigation among Nigerian adults, and Khattak et al. [28] examined Pakistani individuals, all highlighting the existence of ethnic and regional variability in tibial slope morphology. However, these studies did not include inter‐population comparisons, and their clinical applicability remains limited due to methodological heterogeneity. Specifically, regarding the Middle East, AlJuhani et al. [2] evaluated PTS values in a Saudi Arabian population and reported relatively steep slopes (mean PTS angle 13.6 ± 3.4°) compared to international data, suggesting a regional anatomical pattern. However, like other reports in this field, their study was limited by its monocentric design and absence of a matched control group. The current study not only corroborates the findings of these earlier investigations but significantly strengthens the evidence base by providing a direct comparison. These results further support the observation of steeper PTS values in Middle Eastern cohorts, raising important questions about the anatomical and developmental factors contributing to this variation.

These anatomical differences in PTS between populations may be partially explained by environmental, genetic and cultural factors. Daily practices involving frequent deep knee flexion, such as Islamic prayer rituals performed five times a day, may contribute to shaping tibial plateau morphology over time [3]. In many Middle Eastern societies, it is also customary to sit on the floor with knees fully flexed, and in some regions, squatting remains a common posture due to limited access to conventional seating or sanitary infrastructure [4, 17, 22]. These habitual positions may result in functional adaptations that influence PTS development, especially during key growth phases. Pangaud et al. also highlighted how both genetic and environmental stressors can modulate lower limb morphology, including tibial alignment [37]. Such cultural and functional differences may contribute to population‐specific anatomical variability in tibial morphology.

As a secondary observation, this study found a significant difference in graft preference between regions: while hamstring autografts were predominantly used in the Middle Eastern cohort, quadriceps tendon grafts were more common among European patients. This may reflect not only surgical training or regional practice patterns, but also other factors. Although speculative, biomechanical or functional considerations may play a role. For example, graft harvesting from the extensor mechanism, such as quad tendon or BTB, can increase the risk of anterior knee pain, which may impair full flexion or kneeling postures. This may be particularly relevant in populations where kneeling is part of daily activities, such as during Islamic prayer [17, 22, 32]. Similarly, as an exploratory finding, the lower rate of meniscal repair procedures observed in the Middle Eastern group should be interpreted with caution. The difference may reflect variations in timing of surgery, local surgical practice or true differences in meniscal injury prevalence. Given the retrospective design and the absence of standardized timing data, the relative contribution of these factors cannot be determined. These findings may be relevant when interpreting anatomical risk factors in different populations and could be considered in the broader context of clinical decision‐making [5].

In the present cohort, a significantly higher proportion of Middle Eastern patients presented with a PTS greater than 12°. In previous literature, this threshold has been associated with increased risk of ACL reconstruction failure and altered knee biomechanics [44]. Multiple studies have shown that slopes exceeding this value are associated with increased ATT, higher graft loading and elevated rates of graft failure or recurrent instability [5, 11, 18, 19, 34]. The 2022 European Society of Sports Traumatology, Knee Surgery and Arthroscopy (ESSKA) Consensus on ACL revision identifies 12° as a clinically meaningful threshold that may be considered in specific surgical scenarios [9]. Several strategies have been proposed to address the biomechanical burden associated with increased PTS. In particular, the LET procedure has been used as an adjunct to ACL reconstruction in selected high‐risk cases, including those with steep slopes. Biomechanical studies demonstrated reduced graft strain and ATT with LET in steep‐slope models [29, 38]. Clinical studies have also reported reduced graft failure rates and improved stability in patients with PTS > 12° [14, 16, 41], leading to consensus recommendations to consider LET in such settings [40]. In revision scenarios, anterior closing‐wedge high tibial osteotomy (ACWHTO) has been proposed to directly reduce tibial slope and address graft overload [9, 23], with encouraging results reported in selected cohorts [6, 12, 24, 33]. However, no universal agreement exists on the precise cut‐off warranting correction, and debate persists regarding surgical indications [43]. Current approaches increasingly consider PTS as one of several anatomical and clinical risk factors when planning ACL surgery [36]. In the present study, LET rates were similar between the two cohorts, and no ACWHTOs were performed, likely reflecting the primary ACL reconstruction setting. These findings should therefore be interpreted as an anatomical observation within ACL‐deficient cohorts, which may contribute to the broader discussion on how anatomical variability is considered in ACL surgery.

Despite these interesting findings, some limitations must be acknowledged. The inclusion of only male patients, although intended to reduce variability, limits the generalizability of the results to female individuals. Furthermore, the composition of the two cohorts, predominantly Luxembourgish in the European group (64%) and Qatari in the Middle Eastern group (85%), restricts the extrapolation of these findings, which likely reflect regional morphotypes rather than entire continental populations. Moreover, nationality was used as a proxy to define regional groups, as information on ethnicity, long‐term residence or cultural exposure was not available. The retrospective and non‐randomized nature of the study limits the ability to establish causal relationships. Variables such as BMI, detailed time from injury to radiograph and objective activity level were not consistently available and may represent potential confounders. Moreover, PTS measurements were limited to the medial tibial plateau and obtained from standard true short lateral radiographs, which may not fully capture lateral slope variations or asymmetries that could influence knee biomechanics, particularly given the recognized contribution of the lateral slope to rotational instability. However, validated radiographic methods and widely adopted clinical cut‐off values, including those referenced in international consensus statements on ACL revision surgery [9, 40], are primarily based on medial PTS assessment. Finally, no data were collected regarding graft failure rates and ACL revision surgeries, which reduces the clinical applicability of the findings. Regarding this last aspect, this study should be viewed primarily as an anatomical and epidemiological observation rather than a predictor of outcomes. Despite these limitations, the multicenter design and rigorous methodology strengthen the internal validity of the findings. PTS was measured using a standardized and validated radiographic method, with good to excellent intra‐ and inter‐observer reliability. The 1:1 matched cohorts allowed for a meaningful and controlled comparison between two culturally and geographically distinct populations, minimizing selection and demographic bias. To our knowledge, this is the first study to conduct a direct comparison of PTS distribution in ACL‐deficient patients between Europe and the Middle East, offering valuable data in a field where cross‐cultural anatomical comparisons remain limited.

## CONCLUSIONS

ACL‐deficient male patients from the Middle East exhibited significantly steeper PTS angles compared to their European counterparts, with a higher proportion exceeding 12°. This anatomical difference reflects regional morphotype characteristics within the studied cohorts and highlights potential variability in tibial slope distribution across populations.

## AUTHOR CONTRIBUTIONS

*Conceptualization*: Khalid AlKhelaifi, Bashir Ahmed Zikria, Caroline Mouton and Romain Seil. *Methodology*: Khalid AlKhelaifi, Luca Macchiarola and Caroline Mouton. *Formal analysis and investigation*: Iacopo Romandini, Luca Macchiarola, Khalid AlKhelaifi and Caroline Mouton. *Data curation*: Iacopo Romandini, Luca Macchiarola and Caroline Mouton. *Writing—original draft preparation*: Iacopo Romandini. *Writing—review and editing*: Khalid AlKhelaifi, Luca Macchiarola, Bashir Ahmed Zikria, Caroline Mouton, Romain Seil and Alan Getgood. *Supervision*: Romain Seil and Alan Getgood. All authors read and approved the final manuscript.

## CONFLICT OF INTEREST STATEMENT

The authors declare no conflicts of interest related to the present study.

## ETHICS STATEMENT

The study was approved by the Institutional Review Board of Aspetar Orthopaedic and Sports Medicine Hospital, Doha, Qatar (Protocol ID: X202209041). Data collection and handling complied with institutional and national regulations, and the study was conducted in accordance with the principles of the Declaration of Helsinki. All patients provided written informed consent for the scientific use of their clinical and imaging data.

## Data Availability

The data that support the findings of this study are available from the corresponding author upon reasonable request.
